# The Sicker Sex: Understanding Male Biases in Parasitic Infection, Resource Allocation and Fitness

**DOI:** 10.1371/journal.pone.0076246

**Published:** 2013-10-23

**Authors:** Alex Córdoba-Aguilar, Roberto Munguía-Steyer

**Affiliations:** Departamento de Ecología Evolutiva, Instituto de Ecología, Universidad Nacional Autónoma de México, Mexico D.F., Mexico; Institut Pasteur, France

## Abstract

The “sicker sex” idea summarizes our knowledge of sex biases in parasite burden and immune ability whereby males fare worse than females. The theoretical basis of this is that because males invest more on mating effort than females, the former pay the costs by having a weaker immune system and thus being more susceptible to parasites. Females, conversely, have a greater parental investment. Here we tested the following: a) whether both sexes differ in their ability to defend against parasites using a natural host-parasite system; b) the differences in resource allocation conflict between mating effort and parental investment traits between sexes; and, c) effect of parasitism on survival for both sexes. We used a number of insect damselfly species as study subjects. For (a), we quantified gregarine and mite parasites, and experimentally manipulated gregarine levels in both sexes during adult ontogeny. For (b), first, we manipulated food during adult ontogeny and recorded thoracic fat gain (a proxy of mating effort) and abdominal weight (a proxy of parental investment) in both sexes. Secondly for (b), we manipulated food and gregarine levels in both sexes when adults were about to become sexually mature, and recorded gregarine number. For (c), we infected male and female adults of different ages and measured their survival. Males consistently showed more parasites than females apparently due to an increased resource allocation to fat production in males. Conversely, females invested more on abdominal weight. These differences were independent of how much food/infecting parasites were provided. The cost of this was that males had more parasites and reduced survival than females. Our results provide a resource allocation mechanism for understanding sexual differences in parasite defense as well as survival consequences for each sex.

## Introduction

The host-parasite relationship is one of the most widespread and important in nature [1]. Given this, a great deal of research in recent decades has been devoted to understanding how the host's life history traits have been shaped by parasitic defense mechanism investment (reviewed, for example by, [2],[3]). One particular research focus has tried to explain the sex-specific difference in parasite burden [4], which relies on the fact that males are less immunocompetent than females (e.g. [5],[6],[7],[8]). The rationality for why sex difference is as follows: Given that investment in parasite defense is energetically costly (e.g. [9],[10]), mating effort should be traded off against parasite defense but given also that in most mating systems males invest more in mating effort it would stand to reason that there would be a male sex-bias in parasitism ([5],[6],[7],[11]). An example of mating effort is competition for resources that males use to attract females ([8],[12]). Thus, theories of sexual selection and resource allocation explain why males are the “sicker” sex [4].

Despite our theoretical understanding of parasitic biases in both sexes, studies that have assessed their implied resource allocation conflicts are scarce. In particular, the following two issues are lacking to give us a full understanding of how life history and resource allocation can be integrated to understand parasitic sex biases: 1) Theory indicates that such sex biases arise due to a trade off between resource investment of parasite defense vs. mating effort functions in which males invest less to parasite defense and more to mating effort as compared to females [5],[7],[11]. On the one hand, this supposes (at least for some species) a sex-specific shift in resource investment in parasite defense vs. reproductive traits previous to and/or during sexual maturation. Males start allocating more resources to traits related to mating effort compared to females. Although we are aware of ontogenetic transitions whereby individuals invest less towards parasite defense as they reach sexual maturity (e.g. [13], [14], [15]), it is also unclear how this affects resource allocation conflicts in males as compared to females. On the other hand, it is not clear whether females are balancing their resources to favor parental investment traits as opposed to mating effort traits as one would expect. As well, whether a different allocation of resources for both sexes results in a reduced survival rate for males compared to females as a side effect of parasite pressure [4]. There is information regarding differential survival for both sexes on the basis of different parasite defense mechanisms (e.g. [16]), but not in how these survival differences are integrated in the framework of a parasite defense/mating effort trade-off. This information is badly needed to assess the sex-specific fitness costs of potential biases in parasite burden.

**Figure 1:**
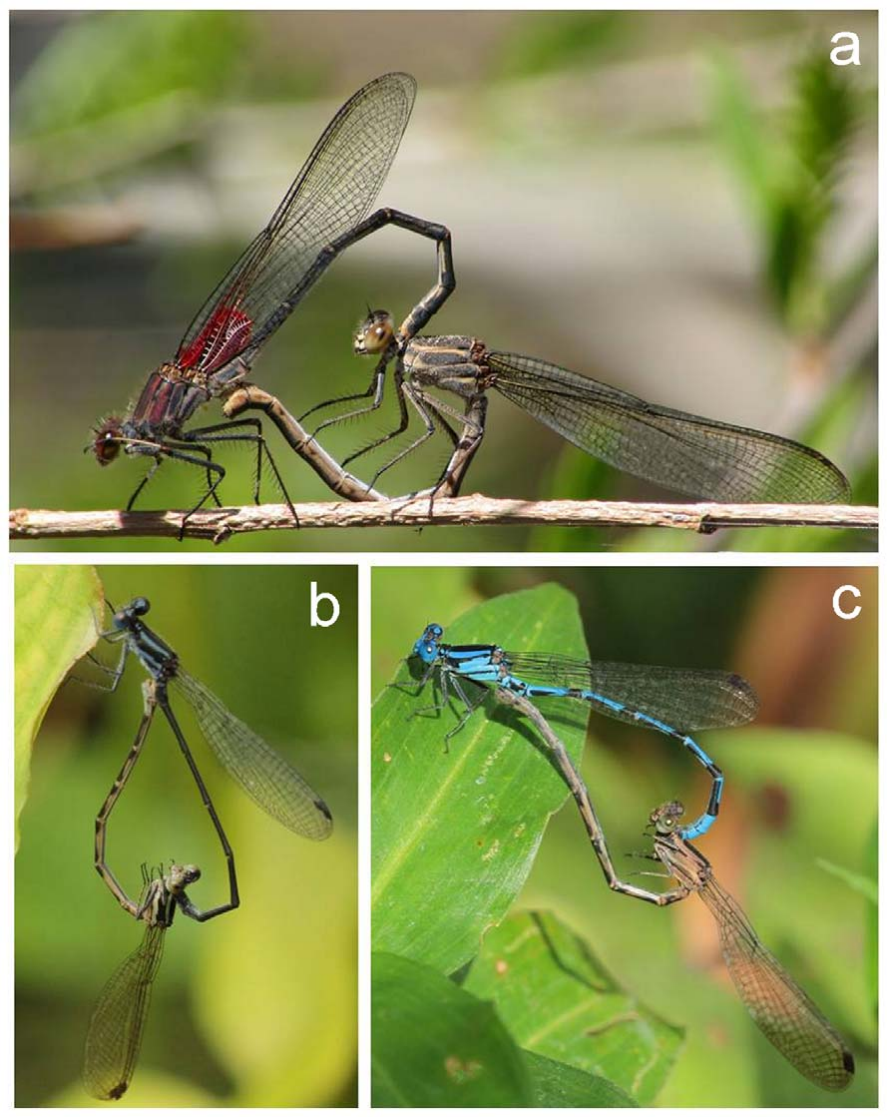
The sicker sex. Three study subjects: a) *H. americana* (photo courtesy, Pierre Deviche); b) *Argia pulla*; and c) *A. extranea* (photo courtesy for both *Argia* species, Benoit Guillon). Males appear above, females below.

In the present work, we have answered the above issues using damselfly insects as host study subjects (Fig. 1) along with two of their main parasitic agents: aquatic mites and gregarines. Damselflies are ideal subject studies for parasitic research for several reasons. In practical terms, they are good for parasite manipulation (reviewed by [17]). Adult damselflies exhibit sex-related biases of aquatic mite and gregarine parasites (reviewed by [17]). These biases have been linked to host's age. While young, non-sexually active adult males and females tend not to show differences in parasite burden (e.g. [18]), a gender bias emerges by the time sexual maturity is reached (e.g. [18],[19]). These biases appear to be related to damselflies' immune ability (which also varies according to season; [20]) where young adults exhibit no sex bias in immunocompetence [21] but sexually active males become less immunocompetent than sexually active females as they mature [19]. However whether this immunocompetence bias is linked to the reason mature adults show sex bias in parasite burden is an unsettled question [17]. Similarly, whether such differential allocation of resources to immune ability affects traits related to mating effort in both sexes is also under studied. A candidate trait to assess this in terms of mating effort, in damselflies is metabolic lipidic fat. The reasons for this is that from adult emergence to maturity, males obtain food resources to construct metabolic fat to be stored in flying thoracic muscles [22],[23]. Unlike females, which use their fat for egg production (in the abdominal region; [24],[25]), males use fat to compete for mates via flying (e.g. [26]). This sex difference in fat use implies that while damselfly males have a larger fat content in the thorax than in their abdomen, the reverse (or a more balanced sharing) occurs for females [25].

The parasites common to adult damselflies are gregarines and aquatic mites [27]. Usually, adult damselflies get infected after ingesting gregarine spores adhered to small prey items such as dipterans (reviewed by [17]). As for the aquatic mites, they attach to the cuticle during adult emergence. The costs of both parasites on damselfly adult hosts are well known in terms of reduced longevity, mating success, body condition, and flying ability (reviewed by [17]). In relation to host's survival, some studies exist in terms of detrimental effects of gregarines but these are mainly correlative (e.g. [28]).

We divided this study into four parts. The first part was to assess whether there are sex biases in parasitic infections. For this we quantified the number of parasites in adult damselfly hosts of both sexes of different ages (to control for potential age-related parasitic differences [29],[30] [31]) and seasons (to control for potential seasonal-related parasitic differences [31],[32],[33]) using both non-manipulated and animal under experimental conditions. The second part was to determine whether parasitic biases are understood on the basis of differential allocation of resources (i.e. higher investment in mating effort traits in males than in females and higher investment in parental investment traits in females than in males). For this, we experimentally manipulated food levels across adult ontogeny in both sexes and evaluated how much energy was allocated to mating effort and parental investment traits. We expected that males would allocate more resources to traits related to mating effort compared to females, but that the reverse would occur for parental investment traits. In order to measure the allocation with regard to mating effort, we assessed thoracic muscle fat, while for parental investment we used abdominal weight (as a proxy of a fecundity related trait; [26]). Third, we investigated whether there was a difference in how males and females trade resources for parasite defense and mating effort. Using a fully factorial design, we manipulated parasite infection and food levels in both sexes at the same age (previous to sexual maturation). While we expected males to invest more in mating effort traits and less to parasite defense, females would be expected to show the opposite trend. Finally, the fourth segment is to measure whether parasite-driven survival is higher in males than in females. We experimentally manipulated parasite infection in adults of both sexes and assessed survival under controlled conditions. We used more than one damselfly species in all experimental segments of the study so that our results are not species-specific. However, we could not control for ancestry effects as the number of species for all parts is not high enough for accurately implementing methods of phylogenetic control.

## Materials and Methods

### Ethics Statement

This research strictly followed the standards of animal use and research stipulated by the Mexican government. A collecting permit issued by the Secretaría de Medio Ambiente y Recursos Naturales (SEMARNAT) was obtained for the collection of specimens used in the study.

### Generalities of insect collection and experiments

Our study was carried out in an approximate 500 m length stretch of the river Amacuzac (18°36′39″ N, 99°10′52″ W, 890 masl). Since parasitic rate can vary according to age in damselflies (see, for example, [34]) it was controlled in all our tests by categorizing three adult ages that have been used in previous studies of age categorization in adult damselflies (e.g. [35]): age 1 are those recently-emerged animals with soft, flexible and colourless body, zig-zag, erratic flight, and do not engage in reproductive activities; age 2 are fully sexually mature animals (as indicated by fully expressed body and/or wing colors and are already engaging in reproductive activities); age 3 are animals whose wings may be broken, and whose bodies are less intense in colour than those of age two, and show signs of pruinescence.

Our experiments required the animals to be left under control-isolated condition prior and during manipulation. Adult damselflies under these conditions reduce their activity and do not die immediately as a consequence of energetic exhaustion (both authors' unpub. obs.). Each animal was allowed to perch while grabbing a stick inside an assay tube that was covered with a humid piece of cotton. All tubes were left inside a dark box at ambient temperature (26–30°C). At the end of the experiment, the animals were preserved in 70% ethanol either for parasite counting and/or fat quantification.

### Assessing gender biases in parasitic rate: damselfly field collection for observational data

We carried out monthly samplings during two different periods: from September 2004 to August 2005, and from May 2006 to April 2007. Each sampling was carried out for two days and during the first week of the month. The following 10 damselfly species were collected: *Argia anceps*, *A. extranea*, *A. harknessi*, *A. pulla*, *A. tezpi*, *A. sp.*, *Enallagma novahispaniae*, *Hetaerina americana*, *Protoneura cara* and *Telebasis salva* (see also Fig. 1). Adult animals of all three age categories and of both sexes were collected. Following collection, animals were preserved for future parasite gregarine and mite counting.

### Assessing gender biases in parasitic rate: experimental data

We manipulated gregarine parasites for experimental infections using the following methodology [36]. For gregarine preparations, we took approximately 20 adults (8–10 males and 8–10 females) of each damselfly age category for each species. We only used these four species (*A. anceps*, *A. extranea*, *H. americana* and *P. cara*) as including all species used for the observational data was too complicated in logistical terms. Furthermore, these four species were the most abundant. Each individual was left in a glassine envelope for 24 hours giving the animals enough time to defecate. The damselfly feces (which contained the gregarine sporozoites) was collected for each individual. The feces of each species were mixed with 400 µl of river water using a vortex for 60 seconds. Using this species-specific source of water, we then provided 1 µl to each experimental animal (the “parasite-treated” group). Experimental groups were determined according to species (*A. anceps*, *A. extranea*, *H. americana* and *P. cara*), sex (males and females) and age (1, 2 and 3). According to this we used at least 20 animals for each combination. We only used these four species as including all those we used for the observational data, was too complicated in logistic terms.

Water containing the parasites was delivered directly through the insect's mouth while the animal was gently held by its wings. We made sure the animal sucked the entire water drop. As a control group (the “parasite control” group), we also provided 1 µl of river water that contained no feces using the same species, sex and age combination as for experimental animals. All animals were left in their assay tubes for 48 hrs with no further manipulation and then preserved for future gregarine counting. This experiment was done once in mid September 2005 and again in mid May 2007 to control for possible seasonal-related differences in parasite burden as these two months show the extreme values in gregarine parasite burden. We did not produce a similar experimental procedure for mites, as we were unable to reproduce the necessary conditions to keep them alive.

### Ontogenetic differences in resource allocation between males and females: assessing mating effort and parental investment traits

In September 2006, we collected adult males and females of all three age categories and of two species, *A. anceps* and *H. americana*, and divided them according to two treatments. We did not use more species due to the logistic complications of this experiment. The two selected species were used because they were the most abundant. One day after collection and for the first treatment (the “fed” group), animals were provided (using dissecting forceps) with as many prey items they could eat as possible during a two-day period. These prey were *Drosophila melanogaster* flies that, before being offered to damselflies, were previously refrigerated for 10 minutes at 5–7°C. At this temperature, flies were still able to move their legs but were incapable of flying. After storage, flies were immersed in tap water for 2–4 seconds. Previous pilot experiments have shown that both fly's leg movements and water immersion facilitate their ingestion for damselflies (both authors's unpub. obs.). Each damselfly was monitored to ensure that they ate each prey item as it may happen that, after chewing it for some time the fly is expelled. Flies were provided three times during each day (so that 6 feeding trials were recorded in total). Animals that did not eat at least 2 flies for each trial were discarded. Animals of the second treatment (the “starved” group), were provided with no food but were manipulated in the same way as those of the fed group. Six hours after the last feeding trial of the fed group, the heads of the animals of both treatments were removed to measure thoracic fat and abdominal weight.

### Differential investment to mating effort vs. parasite defense in both sexes

In June–July 2006, we collected adult males of age 2 of two species, *A. anceps* and *H. americana*. Each species was divided into two treatments according to food provision. The first treatment was given as many adult *D. melanogaster* flies as the damselflies could consume during a two-day period. Flies were previously treated (fridge stored and watered) and provided (using forceps) as indicated in the previous experiment. Animals that did not eat at least 2 flies during 6 feeding trials over the two days were discarded. A control group was treated in the same fashion but no food was provided. After two days, each of these two treatments was divided into two further treatments according to parasite infection. These two parasite treatments consisted of providing animals with 1 µl of river water containing the feces of each corresponding damselfly species and of the same species as indicated above (the water preparation via mixing the feces of 8–10 males and 8–10 females of the three ages that were left in a glassine bag for 24 hrs, with 400 µl of river water), and control animals (1 µl of river water using the same species and sex combination). Thus, we had four groups for each species and sex: fed/parasite-treated, fed/parasite-control, starved/parasite-treated and starved/parasite-control. After parasite delivery, we waited 24 hrs before preserving animals for future gregarine counting and thoracic fat quantification.

### Differences in survival between the sexes

In October 2006, we collected adult males and females of the three age categories indicated above and of the species *A. anceps*, *A. extranea*, *H. americana* and *P. cara*. The experiment commenced one day after collection was completed. The experiment consisted of having two groups of gregarine-challenged and control males (“parasite-increased” and “parasite-control” groups) using the feces-containing river water and “clean” water treatments in the same experimental fashion as we described above when we looked at differences in gregarine parasitism in both sexes. However, the difference here is that after we provided them with river water and while inside their essay tubes, we checked them every hour to see how many were alive until all individuals died. During this revision the animals were not fed. To determine whether the animals were deceased, we made sure that no part of the animal moved.

### Parasite counting

For gregarines, the whole intestinal gut was opened longitudinally using scissors and fine forceps. This was done under a dissecting microscope (Olympus SZH10). Gregarine parasites are easily distinguished due to their whitish appearance. Mites were recorded via two means: a) the number of ectoparasitic mites currently attached to the cuticle and b) the number of cuticle scars left by mites. Both actual mites and scars provide a robust indicator of mite lifetime burden [37]. For both types of parasites, we recorded the abundance (i.e. how many parasites were counted per individual).

### Fat measurement and abdominal weight

The thoraxes of the test subjects were placed in an envelope and left in a desiccator for 24 hrs. The thoraxes were weighed (±0.1 mg), submerged in chloroform for 24 h for fat extraction, re-desiccated and then re-weighed (for similar procedures see [35],[38]). The difference between the initial and the final weights (±0.1 mg) was considered a proxy measure of fat content. Abdominal regions were placed in the desiccator for 24 hrs and weighed (±0.1 mg).

### Statistical analysis

#### Model selection and over-dispersion

In all analyses, global models included all predictor variables as well as their second and third order interactions. In the analyses concerning abundance of parasites, we performed generalized models with Poisson distribution and log link. Models with over-dispersion (more variance than expected) were fitted with a generalized linear model (GLM) using a negative binomial distribution and a log link. For the resource allocation experimental analysis, we assessed thoracic fat and abdominal weight of individuals and performed linear regressions, although fat was log-transformed to improve normality of residuals. In the survival experiment analyses, we fitted parametric models with constant and variable hazard rate distributions (Exponential, Log-logistic and Weibull) and selected the distribution that was the best fit to the data. In all cases we did model selection using values from the Akaike Information Criterion (AIC) to determine the most supported models [39], performed analyses of variance or deviance and estimated the predicted values of specific interactions associated with 95% confidence intervals.

For graphical inspection of results, we used predicted values (except for survival). There are three reasons for this. First, predicted values result from the inference of the most supported generalized linear model, so we can account the difference between levels controlling for other explanatory variables present in the model. Second, the levels of uncertainty in the predicted values can be assessed taking into account the 95% confidence intervals and the probability distribution in the model. Different probability distributions have different ways to assess variance (e.g. binomial, Poisson, negative binomial). And third, predicted values complement the hypotheses tests presented in the tables, because we can graphically assess not only if there difference between levels, but the direction (e. g. which level presents more parasitism) and the magnitude (how different the levels are).

#### Assessing gender biases in parasitic rate: damselfly field collection for observational data and experimental infections

For the observational study of gregarine parasites we assessed the effect of species identity (10 species), sex (male, females), adult age class (1, 2, 3) and year (1, 2) on gregarine abundance in each individual. The global model for gregarine abundance presented an AIC value of 4732.4 while the most supported model had an AIC = 4705.4. Abundance models were fitted with negative binomial distribution since there was over-dispersion (θ = 2.2). In the observational analyses concerning mite abundance, we included the same predictor variables considered for the gregarine analyses. The global model presented an AIC of 3859.5 while the most supported model had an AIC = 3798.2. The GLMs were fitted with a negative binomial model since there was over-dispersion (θ = 0.5264). For the experimental study, the predictor variables were: species identity, experimental infection treatment (parasite-increased, parasite-control), gender, age class (1, 2, 3) and year (1, 2). The global model for gregarine abundance presented an AIC = 3963.7 while the most supported model had an AIC = 3947.5.

#### Ontogenetic differences in resource allocation between males and females: assessing mating effort and parental investment traits

We evaluated the effect of food treatment (fed, starved), gender, age class (1, 2) and species identity (two species) on thoracic fat and abdominal weight of adult individuals. The global model was also the most supported model both for fat content (AIC = −1552) and abdominal weight (AIC = −4662).

#### Differential investment to mating effort vs. parasite defense in both sexes

We evaluated the effect of species identity (two species), food treatment (fed, starved), gender, experimental infection (parasite-treated, parasite-control), fat and wing length on abundance and prevalence of gregarines present in each adult. Global model had an AIC = 1794.5 while the most supported model had an AIC = 1755.3.

#### Differences in survival between the sexes

We assessed survival of adult individuals using treatment (parasite-increased, parasite-control), gender, age class (1, 2, 3) and species identity (4 species) as predictor variables. As the hazard rate was variable, we show parametric models with a Weibull distribution. The global model had an AIC = 6682.9 while the most supported model an AIC of 6882. We estimated Kaplan-Meier survival curves for each species according to sex and experimental infection.

## Results

### Assessing gender biases in parasitic rate: damselfly field collection for observational data and experimental infections

**Figure 2:**
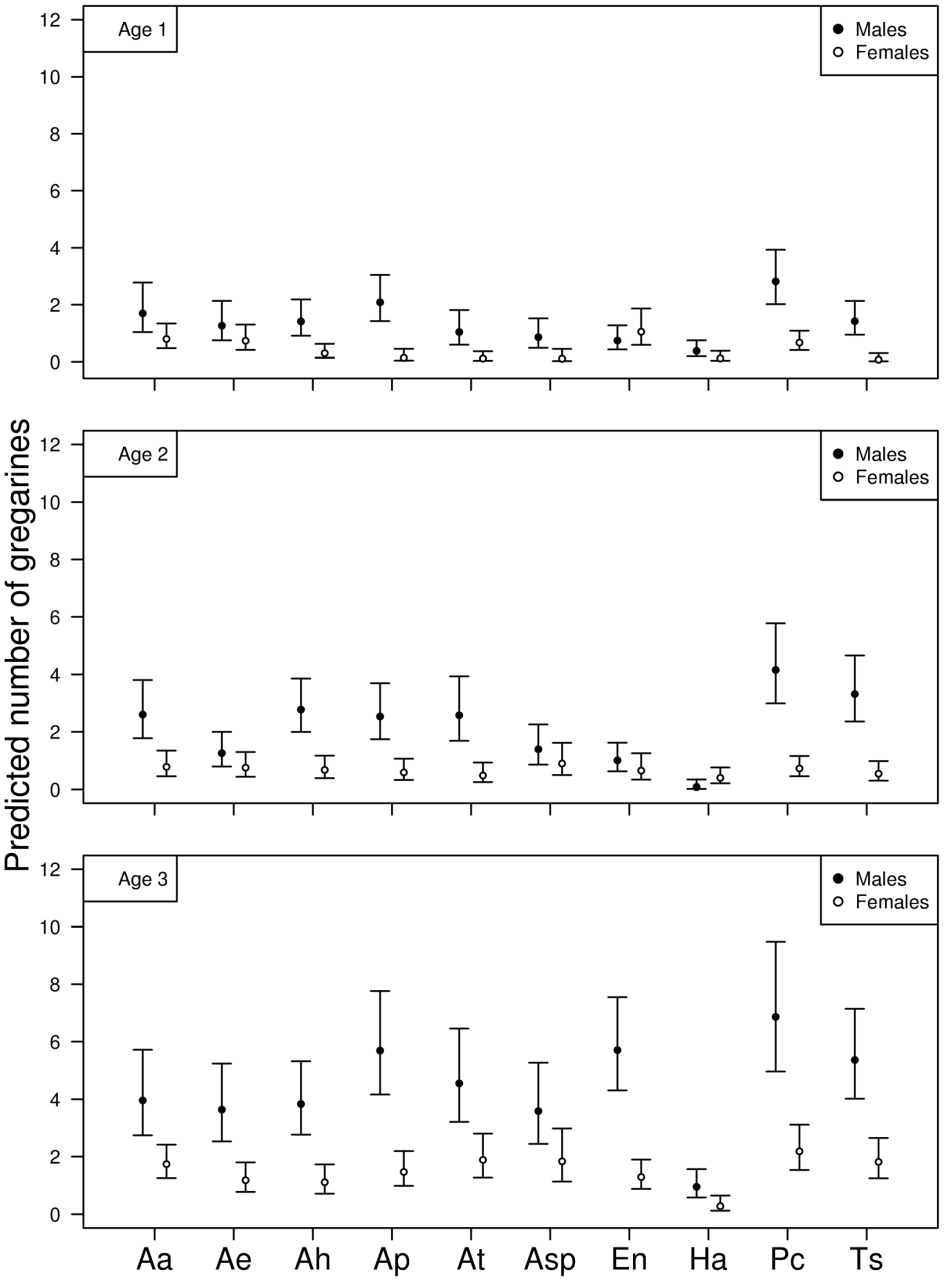
Sex biases in parasitism: observational gregarine data. Predicted gregarine abundance for the observational study according to sex, host species and age class third order interaction. Codes for species identity: Aa = *Argia anceps*, Ae = *A. extranea*, Ah = *A. harknessi*, Ap = *A. pulla*, At = *Argia tezpi*, Asp = *Argia sp.*, En = *Enallagma novahispaniae*, Ha = *Hetaerina americana*, Pc = *Protoneura cara*, Ts = *Telebasis salva.* Error bars depict 95% confidence intervals.

**Figure 3:**
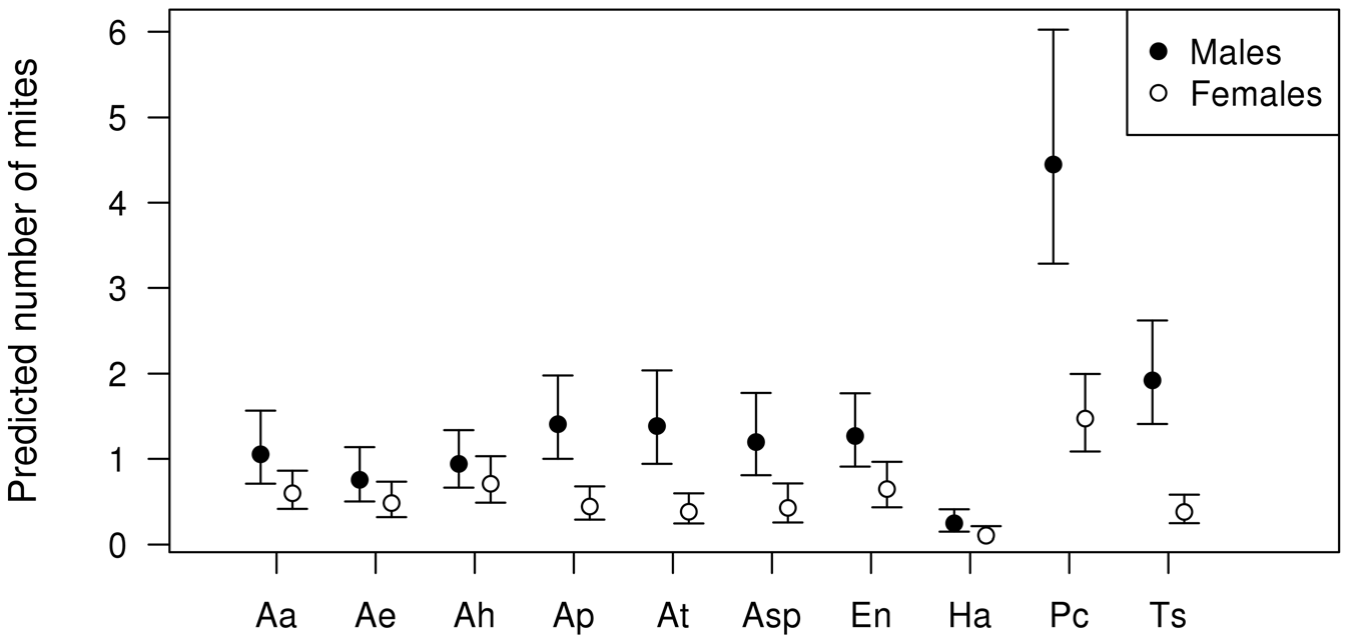
Sex biases in parasitism: observational mite data. Predicted mite abundance for the observational study according to sex and species interaction. Codes for species identity: Aa = *Argia anceps*, Ae = *A. extranea*, Ah = *A. harknessi*, Ap = *A. pulla*, At = *Argia tezpi*, Asp = *Argia sp.*, En = *Enallagma novahispaniae*, Ha = *Hetaerina americana*, Pc = *Protoneura cara*, Ts = *Telebasis salva*. Error bars depict 95% confidence intervals.

Abundance of gregarines (Table 1) and mites (Table 2) differed across species, sexes and ages and their interaction for the observational data set. Males had more parasites than females to varying degree across species (for gregarines see Fig. 2; for mites see Fig. 3). In the case of gregarines, this difference became clearer with older age categories (Fig. 2).

**Table 1:** Analysis of deviance for expected gregarine abundance in the observational study, according to species identity, sex, age year and their interactions.

Predictor	Df	Deviance	Residual deviance	Probability (χ^2^)
Null			2712.2	
Species identity	9	222.4	2489.7	**<0.001**
Sex	1	415.4	2074.4	**<0.001**
Age	2	327.8	1746.6	**<0.001**
Year	1	2.2	1744.4	0.136
Species identity: Sex	9	26.7	1717.7	**0.002**
Species identity: Age	18	36.2	1681.5	0.007
Sex: Age	2	1.4	1680.0	0.487
Sex: Year	1	7.8	1672.2	0.005
Species identity: Sex: Age	18	61.4	1610.9	**<0.001**

**Table 2:** Analysis of deviance for expected mite abundance in the observational study, according to species identity, sex, age and the interaction species: sex.

Predictor	Df	Deviance	Residual deviance	Probability (χ^2^)
Null			1606.0	
Species identity	9	179.4	1426.6	**<0.001**
Sex	1	101.9	1324.7	**<0.001**
Age	2	106.4	1218.3	**<0.001**
Species identity: Sex	9	18.0	1200.2	**0.035**

**Figure 4:**
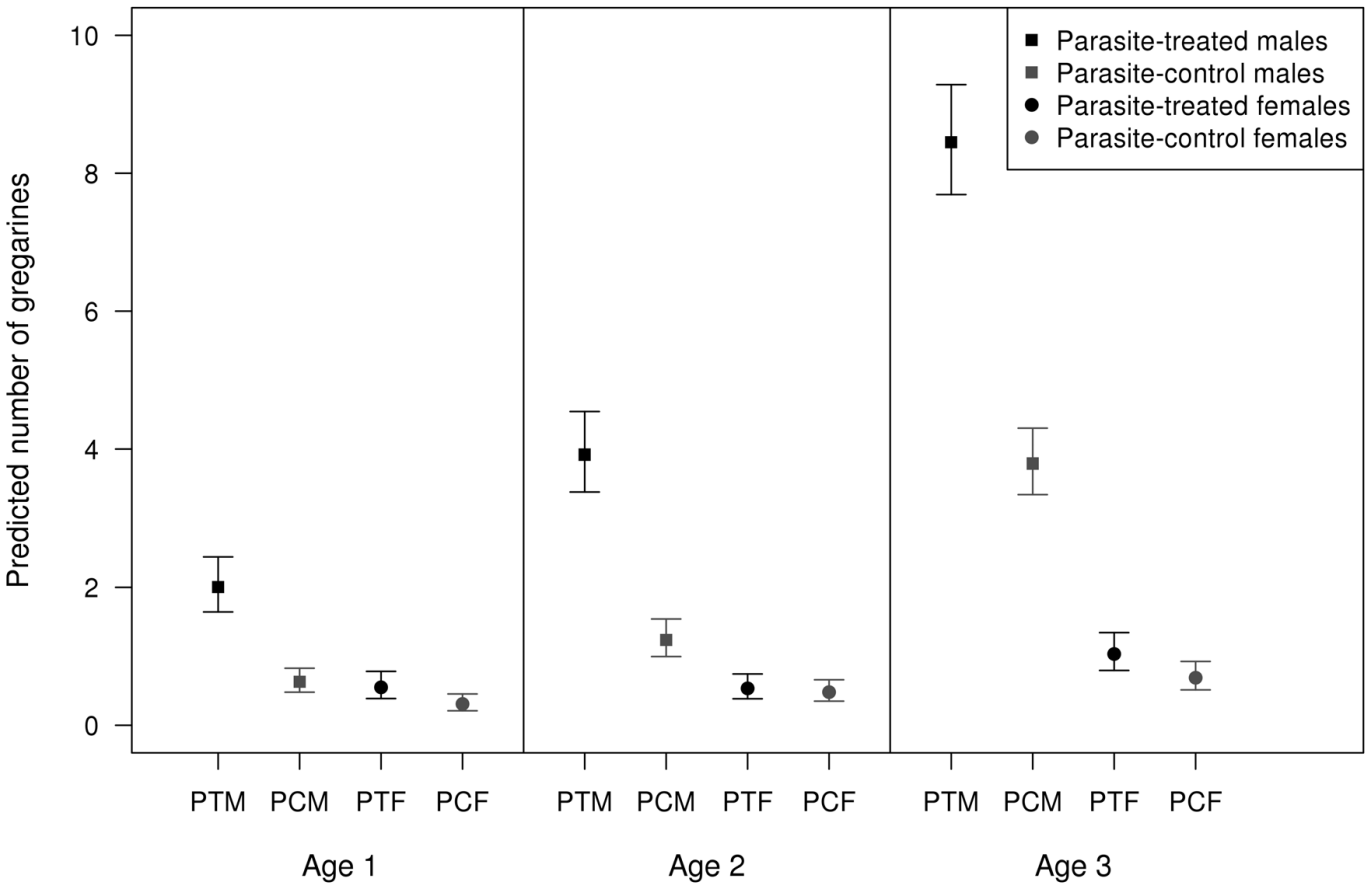
Sex biases in parasitism: experimental data. Predicted gregarine abundance for the experimental study, according to sex, experimental infection and age class. PTM = Parasite-treated males, PCM = Parasite-control males, PTM = Parasite-treated females, PCF = Parasite-control females. Error bars show 95% confidence intervals.

The sex bias difference in the observational data set was corroborated by our experimental study when animals were infected with gregarines (Table 3): experimental infection increased the number of parasites, but males ended up being more parasitized than females and the difference clearly increased as age increased (Fig. 4). There were also differences associated with species, year, and interactions between species identity and experimental infection, sex and age category (Table 3).

**Table 3:** Analysis of deviance for expected gregarine abundance for the experimental study, according to species identity, experimental infection treatment (parasite-treated and parasite-control), sex, age and year and their interactions.

	Df	Deviance	Residual deviance	Probability (χ^2^)
Null			6532.4	
Species identity	3	1562.2	4970.3	**<0.001**
Infection treatment	1	658.4	4311.9	**<0.001**
Sex	1	1423.5	2888.4	**<0.001**
Age	2	862.4	2026.1	**<0.001**
Year	1	5.3	2020.8	**0.022**
Species identity: Infection treatment	3	17.7	2003.1	**0.001**
Species identity: Sex	3	61.6	1941.5	**<0.001**
Species identity: Age	6	14.5	1927.1	**0.025**
Species identity: Year	3	14.1	1913.0	**0.003**
Infection treatment: Sex	1	25.7	1887.3	**<0.001**
Infection treatment: Age class	2	29.3	1858.0	**<0.001**
Infection treatment: Year	1	18.9	1839.1	**<0.001**
Sex: Age	2	99.7	1739.3	**<0.001**
Sex: Year	1	0.7	1738.6	0.390
Species identity: Infection treatment: Age	6	22.4	1716.3	**0.001**
Species identity: Sex: Age class	6	29	1687.4	**<0.001**
Infection treatment: Sex: Age	2	8	1679.5	**0.019**
Infection treatment: Sex: Year	1	5	1675.0	**0.033**

### Ontogenetic differences in resource allocation in males and females: assessing mating effort and parental investment traits

**Figure 5:**
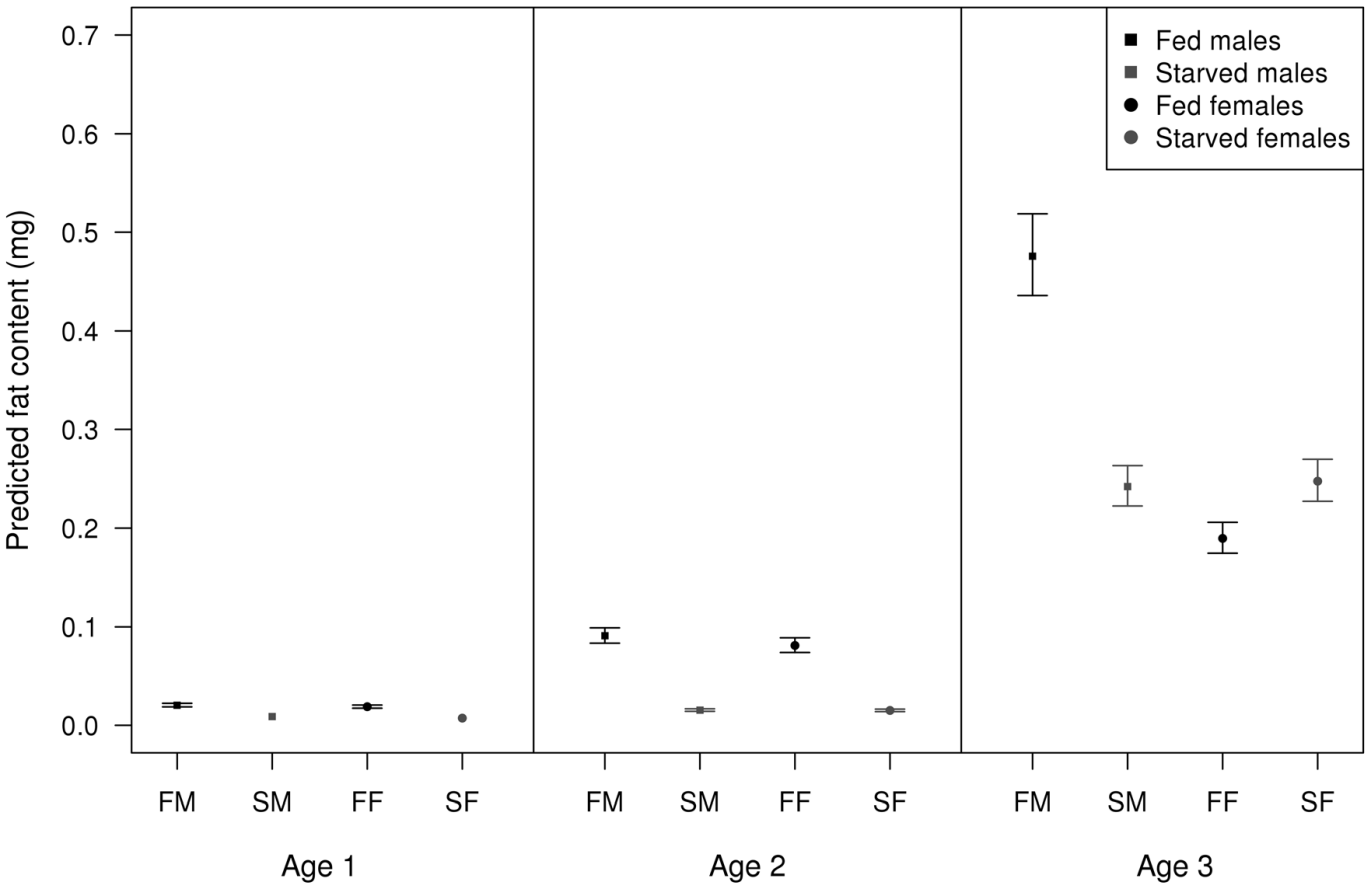
Resource allocation in thoracic fat in both sexes. Predicted thoracic fat content (mg) values of adults according to food treatment, sex and age class triple interaction. Error bars show 95% confidence intervals.

**Figure 6:**
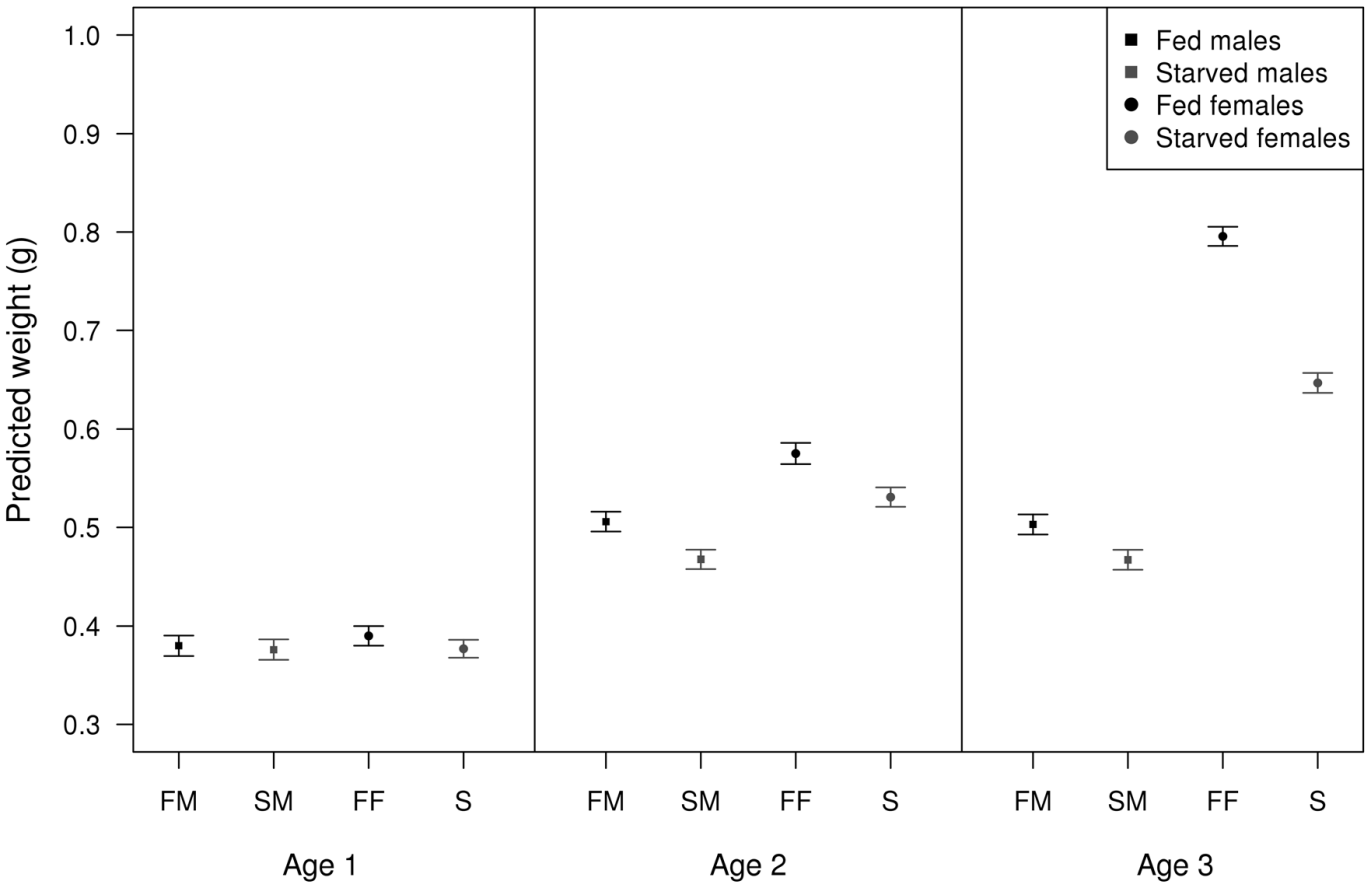
Resource allocation in abdominal weight in both sexes. Predicted abdominal weight (in g) of adult damselflies according to food treatment, sex and age. Error bars show 95% confidence intervals.

Thoracic fat varied according to food treatment, sex, age, species identity and their interaction (Table 4). According to this interaction, initially (at the youngest age), males and females showed marginal differences in how diet resources were diverted to thoracic fat (Fig. 5). However, by the time they reached sexual maturity males allocated considerably more resources to the increase in thoracic fat than females (Fig. 5). Resource allocation to abdominal weight also showed differences according to food treatment, sex, age, species identity and their interaction (Table 5). With respect to this interaction, both males and females increased their abdominal weight in the first two age categories (Fig. 6). However, by the time both sexes reached sexual maturity, females invested substantially more on weight (Fig. 6). This was the case even for the starved females compared to both male treatment groups.

**Table 4:** Analysis of variance of thoracic fat according to food treatment (fed and starved), sex, age and species identity.

	Df	Mean Square	F value	Probability (F)
Food treatment	1	178.1	1598.2	**<0.001**
Sex	1	12.2	109.9	**<0.001**
Age	2	587.1	5269.2	**<0.001**
Species identity	1	3.2	28.5	**<0.001**
Food treatment: Sex	1	4.6	41.4	**<0.001**
Food treatment: Age	2	33.3	298.5	**<0.001**
Food treatment: Species identity	1	11.1	99.4	**<0.001**
Sex: Age	2	2.4	21.8	**<0.001**
Sex: Species identity	1	0.5	4.3	**0.039**
Age: Species identity	2	3.2	29.1	**<0.001**
Food treatment: Sex: Age	2	4.5	40.8	**<0.001**
Food treatment: Sex: Species identity	1	1.9	16.8	**<0.001**
Food treatment: Age: Species identity	2	5.8	52.3	**<0.001**
Sex: Age: Species identity	2	0.9	8.4	**<0.001**
Residuals	695	0.1		

**Table 5:** Analysis of variance of abdominal weight according to food treatment (“fed, starved”), sex, age class and species identity.

	Df	Mean Square	F value	Probability (F)
Food treatment	1	0.46	299.8	**<0.001**
Sex	1	1.71	1111.9	**<0.001**
Age	2	3.17	2057.2	**<0.001**
Species	1	0.55	354.6	**<0.001**
Food treatment: Sex	1	0.09	60.1	**<0.001**
Food treatment: Age	2	0.11	70.4	**<0.001**
Food treatment: Species	1	0.02	11.4	**0.001**
Sex: Age	2	0.85	549.0	**<0.001**
Sex: Species	1	0.02	10.3	**0.001**
Age: Species	2	0.01	7.1	**0.001**
Food treatment: Sex: Age	2	0.05	33.6	**<0.001**
Food treatment: Sex: Species	1	<0.01	3.2	0.076
Food treatment: Age: Species	2	0.04	14.0	**<0.001**
Sex: Age: Species	2	0.02	7.2	**0.001**
Residuals	695	1.07		

### Differential investment in mating effort vs. parasite defense in both sexes

**Figure 7:**
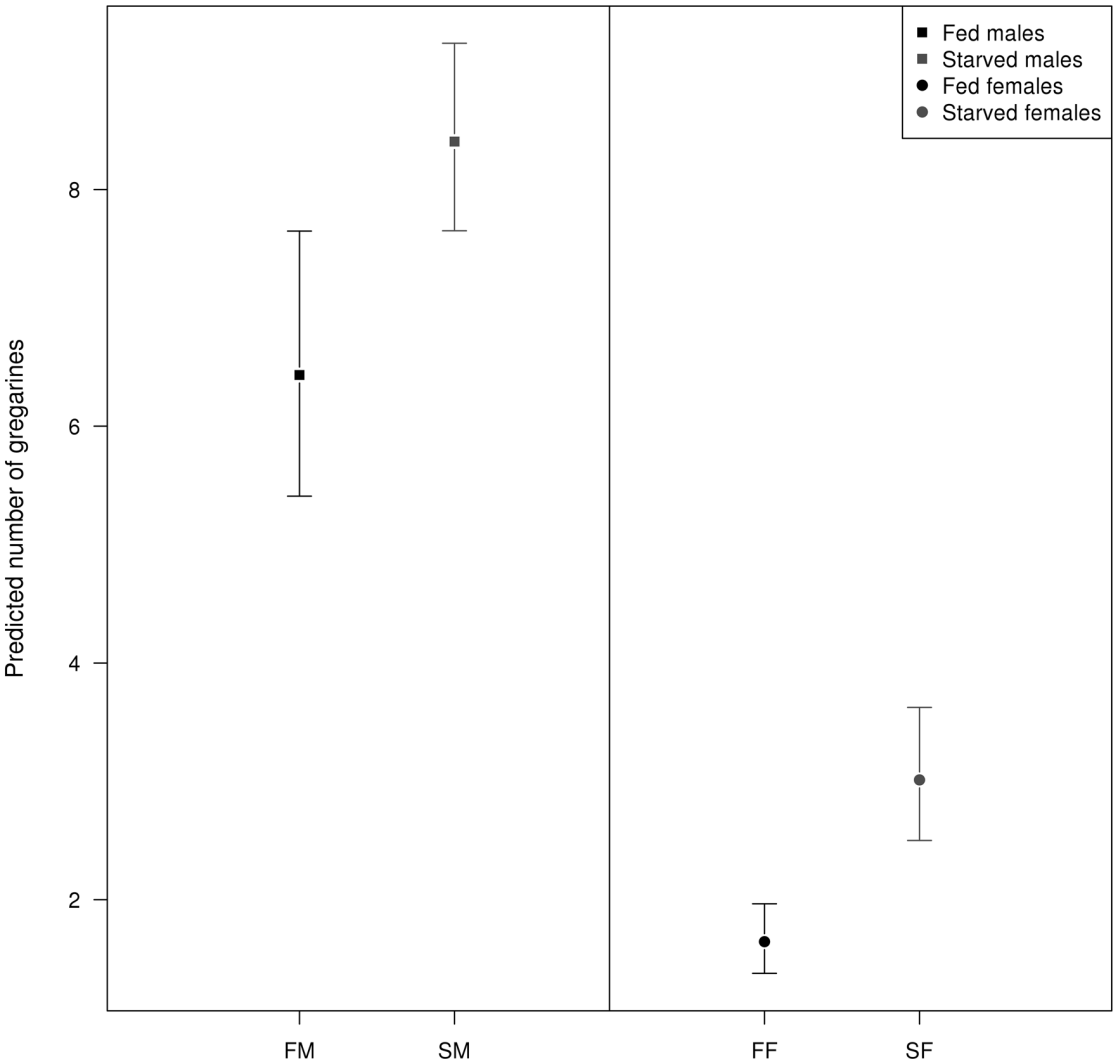
Parasite infection after food stress in both sexes. Predicted gregarine abundance according to sex and food treatment interaction. Error bars show 95% confidence intervals.

**Figure 8:**
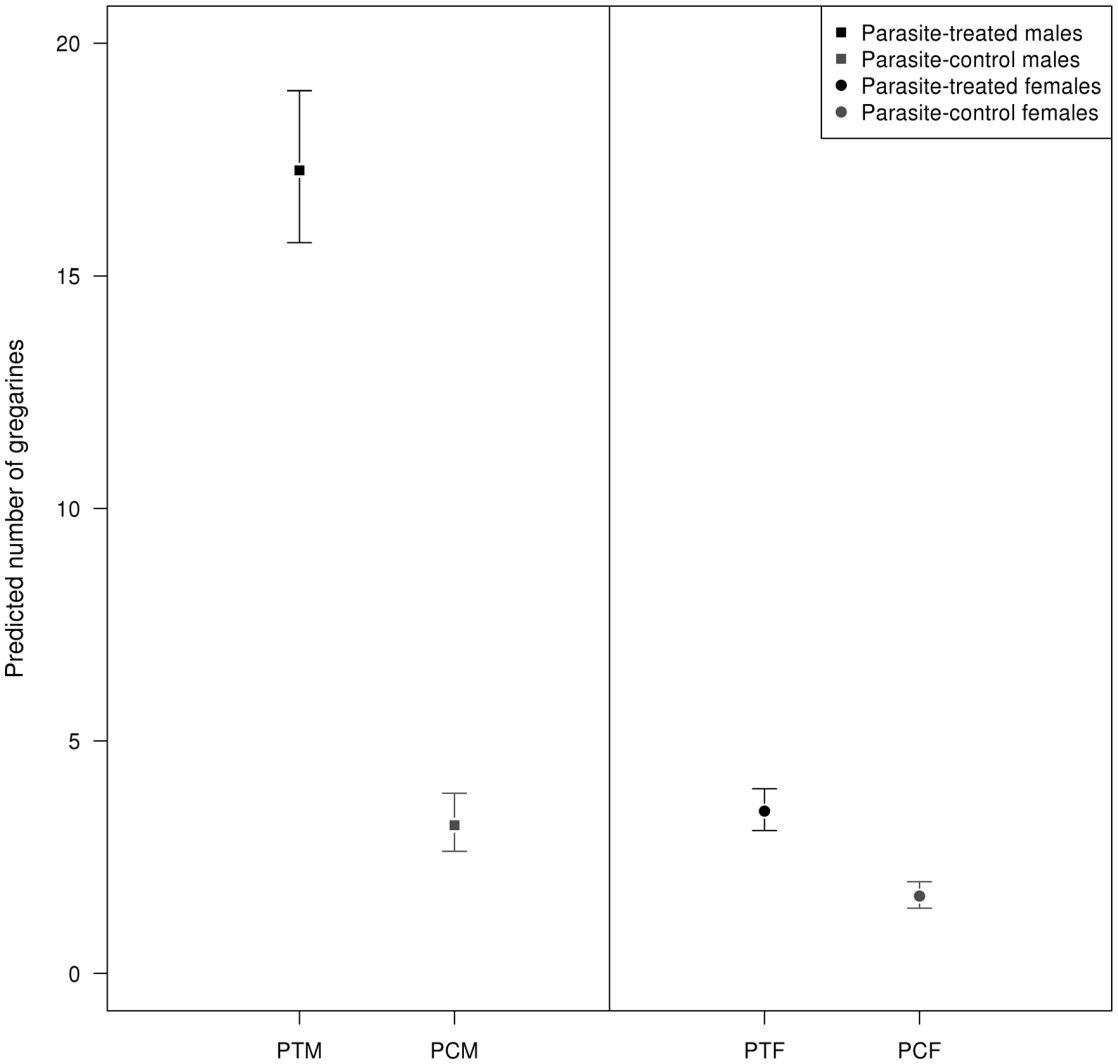
Parasitism rate after experimental infection in both sexes. Predicted abundance of gregarines according to sex and experimental infection interaction. Error bars show 95% confidence intervals.

Gregarine abundance was affected by species, food treatment, sex, experimental infection and wing length (Table 6). Starved individuals presented more parasites than fed animals, where starved males had the highest number of parasites (Fig. 7). Experimentally infected individuals also presented more parasites than control animals, where the males had the highest number of parasites (Table 6, Fig. 8).

**Table 6:** Analysis of deviance of expected gregarine abundance according to species identity, food treatment (fed and starved), sex, infection treatment (parasite-treated and parasite-control), thoracic fat content and wing length.

	Df	Deviance	Residual deviance	Probability (χ^2^)
Null	1		2933.1	**<0.001**
Species identity	1	66.9	2866.2	**<0.001**
Food treatment	1	73.3	2792.9	**<0.001**
Sex	1	1266.0	1526.9	**<0.001**
Infection treatment	1	1050.4	476.5	**<0.001**
Fat	1	15.5	461.0	**<0.001**
Wing length	1	6.4	454.6	**0.011**
Species: Food treatment		0.9	453.7	0.343
Species: Sex	1	5.3	448.4	**0.021**
Species: Infection treatment	1	2.6	445.8	0.105
Food treatment: Sex	1	6.0	439.8	**0.014**
Food treatment: Infection treatment	1	0.8	439.0	0.383
Sex: Infection treatment	1	79.1	359.9	**<0.001**
Sex: Fat	1	0.1	359.8	0.778
Sex: Wing length	1	0.1	359.7	0.716
Fat: Wing length	1	1.3	358.4	0.263
Species: Food treatment: Sex	1	3.4	355.1	0.066
Sex: Fat: Wing length	1	3.9	351.2	**0.048**

### Differences in survival between the sexes

**Figure 9:**
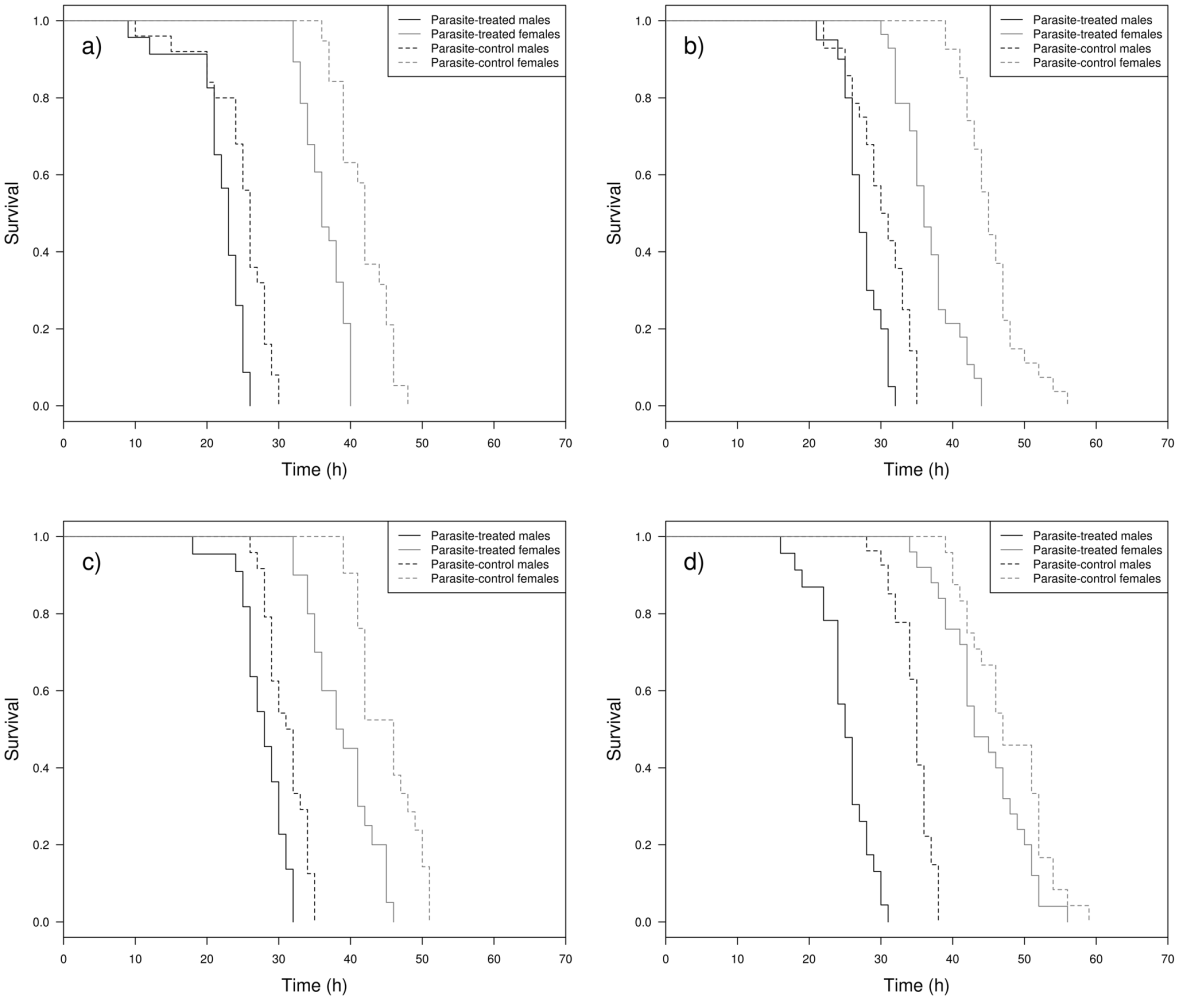
Survival and sex after infection. Kaplan-Meier survival curves according to species, sex and experimental infection interaction at age 3. a) *Argia anceps*, b) *A. extranea*, c) *Hetaerina americana* and d) *Protoneura cara*.

**Figure 10:**
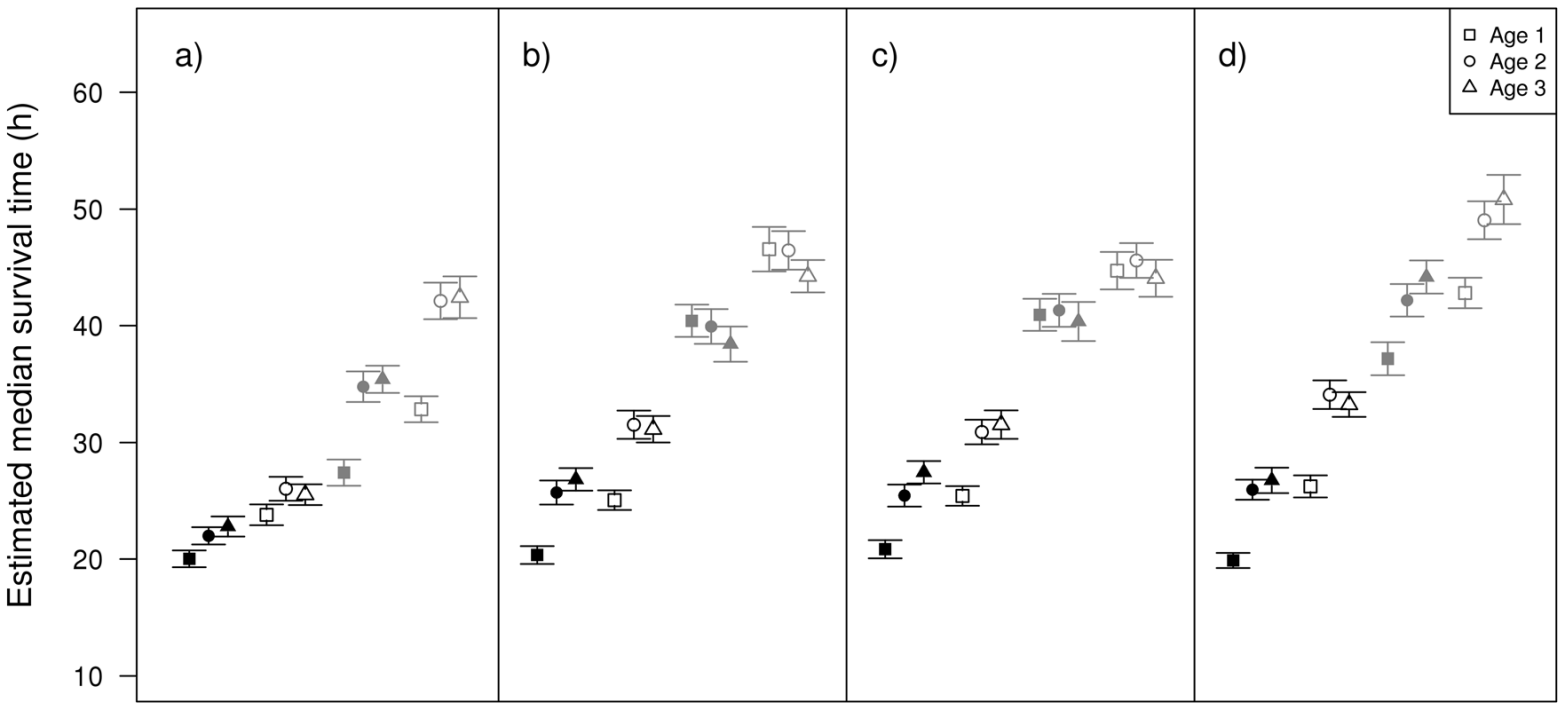
Expected survival and sex after infection. Expected median survival time according to species, sex, infection treatment and age. Black symbols = males, gray symbols = females. Filled symbols = parasite-increased, empty symbols = parasite-control. Error bars show 95% confidence intervals. a) *Argia anceps*, b) *A. extranea*, c) *Hetaerina americana* and d) *Protoneura cara*.

There were differences in survival rate according to infection treatment, sex, age, species identity and most of their interactions (Table 7). In relation to what we predicted, animals with higher parasite levels had a lower survival rate than parasite-control animals for all species (Table 7; Figs. 9 and 10). However, males had a lower survival rate than females and infected males showed the lowest survival rates (Fig. 9). These differences were age- and sex-dependent with young ages and males showing lower survival rates than older individuals and females, respectively (Fig. 10).

**Table 7:** Survival analyisis concerning the effects of infection treatment (parasite-treated and parasite-control), sex, age and species identity.

	Df	Deviance	Probability (χ^2^)
Null			
Infection treatment	1	86.60	**<0.001**
Sex	1	1286.81	**<0.001**
Age	2	198.71	**<0.001**
Species identity	3	310.89	**<0.001**
Infection treatment: Sex	1	23.35	**<0.001**
Infection treatment: Age	2	7.17	**0.028**
Infection treatment: Species	3	18.82	**<0.001**
Sex: Age	2	78.56	**<0.001**
Sex: Species identity	3	9.67	**0.022**
Age: Species identity	6	43.51	**<0.001**
Infection treatment: Sex: Age	2	4.23	0.121
Infection treatment: Sex. Species identity	3	11.45	**0.009**
Sex: Age: Species identity	6	147.48	**<0.001**

## Discussion

Our results support the prediction that males are higher propensity for parasitization than females. Despite recent reviews concluding that males are the sicker sex, there are some inconsistencies to this claim (e.g. [3],[40],[41]). In fact, even using only damselfly species there was no clear pattern of sex bias although all of the studies were non-experimental (reviewed by [17]). In damselflies, this lack of a bias pattern may be caused for a number of reasons including that age is positively correlated with parasite accumulation (e.g. [34]) and seasonal variation in either host's immune response [30] or parasite exposure (e.g. [42]). Indeed, looking at our observational data, the male bias pattern is age dependent at least for some species: young adults tend to show less gender differences in parasitism. This may be partly explained by a the immune ability of young adults in both sexes and how it changes with age [21],[43],[44]. This sex dependent pattern has also been detected in other insects whose sexes, previous to becoming sexually active, do not exhibit differences in immunocompetence (e.g. [45]). The sex bias was also explained by year of experimental infections. Yearly variation in resistance in both sexes has been interpreted as evolutionary response to the intensity of parasitism in odonates [46]. How such yearly variation in parasitism affects each sex's allocation of resources to parasite defense and fitness has not been explored.

Parasitism was related to adult survival according to our experiments. Previous non-experimental studies have shown that in damselflies and other insects, natural parasites can impair their hosts' survival rates (reviewed by [3]). In proximate terms, gregarines may exert several negative effects on odonate hosts: a) a reduction in host's fat stores possibly by sequestering the host's food resources for gregarine development and reproduction [34]; b) a reduction in male muscle performance during aerial competition via disrupting the metabolic pathways by which thoracic fat is used during muscular activity [47],[48] and, c) breakage of the host's intestinal tissue which may allow other infective pathogens to invade the rest of the animal body [49] (following a similar claim in Trichoptera larvae see [50]). How damselfly males and females differ in their ability to deal with gregarine effects is hard to explain. Given that our survival experiments were carried out in captivity, there are implications that explanations such as the reduction of male muscle performance may not apply in this study, as our animals did not have to fly at all. However, explanations such as reduction of host's fat stores and breakage of intestinal tissue did apply. For example, it may be that males are more susceptible to having their fat resources sequestered by gregarines than females. This makes sense given that thoracic fat seems a more condition-dependent trait in males than females. If part of this fat were used for surviving effects, then males would become more likely to die in case of an acute gregarine infection as compared to females. In the case of the gut tissue breakage, one explanation is that males have less resistant tissues than females. These effects and others need to be explored in greater detail in future research.

From a resource allocation perspective, our study provides cues of how each sex solves the problem of dealing with parasites differently to mating effort and parental investment pressures. Selection for fecundity has been well established in insects (e.g. [54]), so that females possibly trade off their resources between mating effort and parental investment traits as our findings suggest. Theory has emphasized that since males are under more pressure to deviate more resources to mating effort traits than females [5],[6],[7],[8],[11], males may become more prone to be parasitized than females. Several studies have, for example, found that investment in testis size (a proxy of mating effort) may result in males being less immunocompetent [51], [52]. In our work, as a mating effort trait for males we used thoracic fat, a trait that has evolved with marked sex-based differences in damselflies due to intense selection on males during mate competition [53]. On the one hand, our results confirm this pattern but elucidate an age-dependent effect, as neither sex seems to differ in fat allocation early in their adult life. This ontogenetic difference may be that previous to sexual maturation, both damselfly sexes face similar natural selection pressures and so the fat allocation does not differ [53]. However, by the time both sexes reproduce, males require a large thoracic fat volume to endure male-male competition [53]. This is not the case for females, as females need to invest more on fecundity related traits [36]. This need is quite clear even with starved females as they showed a proportionally higher investment in abdominal weight as compared to fed and starved males. Selection for fecundity has been well established in insects (e.g. [54]), so that females possibly trade off their resources between mating effort and parental investment traits as our findings suggest.

One consequence of parasite defense in the sexual differences in resource allocation was revealed by our experimental combination of food and parasitism stress-factors. Fed males with no parasite increase invested less in parasite defense (as assessed by gregarine burden) compared to females in the same situation. This implies that no matter how stressful the environment is, males will become more susceptible to infections than females as they age. These findings support the theoretical expectation of males being the “sicker” sex [5],[6],[8],[11]. However, it speaks to the fundamental yet under studied mechanism of resource allocation [4]. Also, our results fit with recent theoretical advances in immunity and life history trade offs, which indicate that the intensity of sexual selection will always render males to become the sicker sex [8],[11],[13]. Despite the fact that even when our subjects include species with potentially different varying sexual selection intensity for the few species that this is known (e.g. lekking species, *H. americana*, [20]; resource defence polygyny, *P. cara*, [55]; non-territorial, scramblers, *T. salva*, [56]) a strong sexual dimorphism was shown in parasite burden by the time adult hosts are sexually mature. In other words, the sicker sex principle is well illustrated in a plethora of mating systems.

Our observations and experiments also reflected significant differences according to species (for parasite abundance, resource allocation and survival) and year (for experimental infections only). Three reasons can explain species-specific differences. The first is the varying energetic requirements acting within each species but differing among species. One example is the case of damselflies with female polymorphism in which the male-mimicking morph shows a higher parasite burden compared to other female morphs. This is presumably [57] due to resource-allocation cost of becoming a good male mimic [58], [59]. In fact, one of our study species, *E. novahispaniae*, has two polymorphic females. A second reason is species-specific differences in parasite exposure depending on habitat and season [e.g. 60]. In fact, several of our study species differ in the microhabitats preferred by adults. One case is *T. salva* where more open vegetation areas are favored as compared to *H. americana*. These differences in vegetation may be linked to gregarine presence and abundance [61]. A third reasons is that species differ in parasite defense ability, an ability that has been shown in dragonfly species [62]. In terms of yearly cycle differences in parasite burden, prevalence and intensity of parasite infection have been associated to damselfly resistance and both traits are known to vary with by year. This implies correlated cycles of host resistance and parasite abundance [63]. The yearly cycles were not taken into consideration in this study but would be useful for other aspects of parasite burden and impact in a future study.

Finally, one key issue is possible common ancestry effects in our study. Our subject species are very different in morphology (e.g. sexual size dimorphism: males larger than females in *H. americana* and females larger than males in *T. salva* [64]) and mating systems (see above) which could shape the energy needed for traits such as those related to parasite defense [65]. Despite this and contrary to what one would expect if ancestral effects were the main explanation, it is notable that the sicker sex principle is common in each species.
